# Supplementary material for: Are carbon emissions trading and green financial instruments synergistic? -Comprehensive quantitative research based on content analysis
Source: PLoS One. 2024 Mar 7;19(3):e0298601. doi: 10.1371/journal.pone.0298601 (PMC10919601; doi:10.1371/journal.pone.0298601)
Supplement: S1 Data — (ZIP) [file pone.0298601.s001.zip › Data/地级市Z_F工作报告.zip!/╕╩╦α/└╝╓▌╩╨/└┤╫╘╣1⁄2╓┌║┼í╛┬φ┐╦╩2╛▌═°í┐_2015.docx]

各位代表：

　　我代表市人民政府向大会作工作报告，请予审议，并请市政协委员和其他列席人员提出意见。

　　一、2014年工作回顾

　　过去一年，面对错综复杂的国内外发展环境，在省委、省政府和市委的坚强领导下，全市上下按照“明地位、强责任、作表率、出大力”的新要求，全力实施“3341”项目工程和“1236”扶贫攻坚行动，统筹做好稳增长、调结构、促改革、治污染、畅交通、惠民生等重点工作，努力实现市十五届人大四次会议确定的目标任务。

　　——综合经济实力持续提升。全市实现生产总值1913.5亿元，增长10.4%。全社会固定资产投资1610.7亿元，增长22.3%。社会消费品零售总额944.9亿元，增长12.7%。公共财政预算收入152.33亿元，增长22.36%。进出口总额占全省的52.7%。12项主要经济指标在全省市州的排名均有前移，8项排在前3位。

　　——经济结构更加优化。第三产业增加值增速高于第二产业2.7个百分点，三次产业结构调整为2.8∶43.34∶53.86，第三产业比重提高2.82个百分点。战略性新兴产业增加值达到153亿元，增长11%，占生产总值的比重提升到8%。非公有制经济增加值占全市生产总值的一半。

　　——人民生活水平稳步提高。城镇居民人均可支配收入23030元，农民人均纯收入8067元，分别增长10.9%和13.4%。新增城镇就业11.56万人，农村劳动力转移就业25.03万人。104个村整体脱贫，减少贫困人口12万人。

　　——大气环境质量明显改善。实现了“一年全面攻坚、两年初见成效、三年明显改善”的目标，城区优良天数达到313天，比上年增加14天，尤其是优质天数增加11天，轻度以上污染天数减少10天，成为兰州有监测记录以来优良天数最多的一年。治理大气污染的“兰州经验”得到国家肯定。

　　——城市开放度不断提升。西客站和兰新高铁开通运营。新开通兰州至迪拜、第比利斯、新加坡3条国际航班，中川机场升级为国际客货运机场，新航站楼建成投运，旅客吞吐量突破700万人次，出入境旅客突破10万人次。在兰州国际马拉松赛、中国金鸡百花电影节等赛事节会的带动下，国内外来兰游客达到3400万人次，增长30.13%。

　　——争取政策支持再获新突破。国务院批复设立兰州新区综合保税区。获批创建全国节能减排财政政策综合示范城市，是多年来一次性争取国家资金支持最大的政策平台。还争取到全国质量强市示范城市、国家城市矿产示范基地、国家电子商务示范城市、信息惠民国家试点城市、兰白科技创新改革试验区等政策平台。省委、省政府出台《支持兰州转型跨越发展的意见》。向上争取各类资金37.72亿元。

　　一年来，我们突出省会中心城市特点，加大经济结构调整力度，推动产业转型升级。大力扶持现代服务业发展，开展“电子商务发展年”活动，引进中国网库建设甘肃电商谷，淘宝“特色中国·甘肃馆”落户兰州，与阿里巴巴达成合作意向，建成三维商城等本土电商平台，跨境电子商务实现零的突破，电子商务交易额增长61.2%。瑞鑫、毅德、北龙口等大型物流园区加快建设。万达广场等城市综合体投入运营。民生银行兰州分行挂牌营业。促进文化与旅游融合发展，大兰山、仁寿山、河口古镇、青城古镇、金城公园、石佛沟等景区建设呈现新亮点，旅游收入达到268.64亿元，增长30.09%，实现文化产业增加值51.3亿元，增长25.5%。坚持传统产业改造和新兴产业培育两手抓，实施“3+7”工业倍增计划，打造石油化工、有色冶金、装备制造三大千亿产业链和电子信息、生物医药、新能源、环保、建材、烟草、食品及轻工七大百亿产业链，新建成工业项目117个，新增产值193亿元。兰石重装成功上市。8家企业入围全省战略性新兴产业骨干企业，新增高新技术企业10家。实施创业带动就业“三个20万”政策，制定了“万企计划”，扶持初创微型企业7571户，带动就业5.7万人。非公有制经济实现增加值965亿元，增长28%。

　　一年来，我们围绕国家赋予的战略定位，坚持高标准建设、新模式开发、大项目支撑，全力推进兰州新区开发建设。基础设施建设快速推进，道路通车总里程230公里，中马铁路建成投运，兰秦快速通道全线竣工，兰州至中川城际铁路加快建设，核心区100平方公里的水、电、路、气等基础设施基本建成。以六大产业片区建设为核心，着力加大招商引资和项目建设力度，实施产业项目112个，科天化工项目建成投产，正威铜加工项目加快建设；实施“出城入园”项目55个，兰石集团、青岛啤酒等8个项目建成投产。综合保税区10个必建工程加快建设，引进厦门优传、金合智能液压等24个项目，总投资24.6亿元。统筹推进公共服务设施建设，兰州实验幼儿园新区分园建成开园，兰炼一小、兰州十一中新区分校、中小学综合实践基地及急救中心、残疾人康复中心、民生医院加快建设。新区完成固定资产投资435亿元，增长44.5%，生产总值达到96.36亿元，增长33.12%，正在成为全市经济发展新的增长极。高新区、经济区生产总值分别达到185.22亿元和182.95亿元。

　　一年来，我们在国家和省委、省政府的支持下，以更大力度推进大气污染治理整体战攻坚战。争取到中央和省级专项资金3.87亿元。完成100万平方米未供暖建筑改造。治理改造城区剩余870蒸吨燃煤小锅炉，实施2108蒸吨燃气锅炉余热深度利用项目。完成231个工业深度治理项目。出台城市道路挖掘管理办法，对所有建筑工地实行电子眼全程监控。淘汰黄标车和老旧汽车6.4万辆。严格环保执法,对环境违法行为予以严肃查处并公开曝光。二氧化硫、氮氧化物、化学需氧量、氨氮减排量分别完成年度计划的201%、113%、445%和295%，二氧化硫、化学需氧量、氨氮提前超额完成“十二五”减排目标。大气污染治理改善了环境质量，优化了发展环境，提升了城市形象，提高了群众健康水平，进入冬季供暖期的三个月，全市城乡居民呼吸系统疾病就诊病例和就医费用，同比分别下降8.16%和20.49%。

　　一年来，我们立足城市可持续发展，加大城市规划建设管理力度。编制中心城区抽疏规划和城郊片区规划，实现中心城区单元控详规划全覆盖。完成城市六大出入口和黄河风情线规划设计。轨道交通1号线一期工程全线开工建设，累计完成投资76.65亿元。加快完善“139”路网体系，南山路29公里实现通车，北环路加快建设。天水路下穿读者大道和白银路上跨安定门什字工程竣工通车。新建人行过街天桥20座。深安黄河大桥建成通车。开通运营公共自行车租赁系统和黄河水上公交。实施81条道路单向通行，形成22组交通微循环。积极谋划城市长远供水问题，开工建设城市水源地项目。完成雁滩南河道排污口截流治理工程。开展黄河风情线综合整治，完成中山桥至体育公园治理改造。新增城市绿地232.25公顷。启动城区地下管网普查工作。实施大规模棚户区改造，申请国家开发银行授信额度212亿元，到位71.2亿元，新开工建设棚户区和保障性住房2.9万套。启动“智慧兰州”建设，开通三维服务网。出台向社会力量购买服务《实施办法》和《指导性目录》，在黄河风情线卫生保洁等领域逐步推广实施。

　　一年来，我们把人力物力和财力向农村倾斜，扎实推进扶贫攻坚和“双联”行动。全年整合投入各类帮扶资金5.92亿元，实施项目941个，超额完成贫困村整村脱贫任务。实施设施农业、肉羊产业、土地流转、环境整治“四个千万”扶持政策，新增设施农业1.1万亩、肉羊24万只、土地流转18万亩，建成10个省级、20个市级美丽乡村示范村和85个环境整洁村。集中推进榆中北山中药材产业发展，新增种植面积5万亩，累计达到18万亩。开展农民技能培训10万人次。建成596公里通村公路，建制村通畅率达到93.1%。建成20个新型农村集中居住区，完成危房改造5000户。解决农村10.91万人饮水安全问题。建成农村文化活动室100个、全民健身场地207个、“乡村舞台”310个、城乡老年人日间照料中心100个、标准化卫生室32个。

　　一年来，我们顺应群众期盼，强化问题导向，切实加强民生服务和保障工作。完成市列为民兴办实事24件、省列实事22件。开展“十大就业创业专项行动”，城镇登记失业率控制在1.73%。积极创建“无欠薪”城市，因欠薪引发的群体性事件下降21%。城乡居民基础养老金、城镇居民医疗保险财政补贴和企业离退休人员月平均养老金分别提高到85元、320元和1650元。城乡居民社会养老保险参保率96.8%，7.8万名完全失地农民纳入养老保险。城市低保补助五区每人每月由407元提高到468元，三县每人每月由306元提高到352元。农村低保每人每年由1920元提高到2210元，五保补助每人每年由2800元提高到3310元。启用新农合“一卡通”，新农合参合率达到98%，人均政府补助由290元提高到330元。发放住房公积金贷款32.56亿元，解决1.2万户家庭购房资金需求。

　　积极发展各项社会事业。对城区住宅小区中小学幼儿园配建情况进行系统调查摸底，制定了三年落实方案。创建标准化学校121所。继续实施校安工程，改建C级危房21.7万平方米。发放农村义务教育阶段学生营养早餐补助6190万元。完成科技工作厅市会商事项26项，成功举办首届兰州科技成果交易周。推进科技惠民示范工程建设，支持了中药材规模化种植、动植物新品种引进培育、农产品深加工等项目。建成开放“红星照耀中国”主题展馆和兰州战役纪念馆。公益性文化设施全部实现免费开放。市公众安全就医服务平台建成运行。公共场所控制吸烟条例正式实施。

　　全力维护社会和谐稳定。积极践行社会主义核心价值观，树立了“兰州好人”等文明创建品牌，“凡人善举、和你一起”活动入选中宣部创新案例。成立公共安全应急管理专家咨询委员会，修订完善各项公共突发事件应急预案。食品药品安全攻坚战取得显著成效，食品安全电子追溯体系建设走在全国前列，九州“主食厨房”食品加工园区部分建成启用。凝炼了“品质如兰、秉德之州”的城市质量精神，电梯等特种设备安全运行得到进一步强化。“12345”民情通服务热线办理群众反映诉求66万多件，回复人民网和市政府网站留言5191条。开通运行网上信访大厅。“六五”普法获全国中期先进城市称号。民族宗教工作“三个共同、两个提升”活动深入开展。“平安兰州”建设卓有成效，八类主要刑事案件下降22.5%，命案侦破率达到100%。安全生产四项指标全面大幅下降，安全生产形势稳定好转。

　　一年来，我们坚持从转变政府职能抓起，探索和落实重点领域改革举措。深化行政审批制度改革，取消行政审批事项20项，下放9项，调整22项，市级政府部门行政审批事项减少到203项。编制和公布政府部门权力清单1989项、企业投资项目管理负面清单177项。市政务服务中心推行重大项目并联审批和代办服务模式，新设立企业“三证合一”实现当日办结。健全完善公共资源交易系统，完成入场交易项目1461项，交易金额248.76亿元。完善预算编制、执行和监督管理，实现部门预决算全公开。出台政府性债务管理暂行办法及配套制度，建立了稳定的偿债准备金机制。实施工商登记制度改革，新登记注册各类企业2万多户，比上年翻了一番多。推进国资国企改革，整合优化国有企业经营性资产、非经营性资产和国有股权，组建了兰州投资（控股）集团。顺利完成全市第三次全国经济普查工作。

　　一年来，我们努力走开放带动发展的路子，积极参与丝绸之路经济带建设。在全国省会城市中率先加入上海合作组织睦邻友好委员会。成功举办丝绸之路经济带（兰州新区）发展高层论坛、聚焦丝绸之路·500强企业高峰论坛等系列活动。在韩国首尔举办向西开放战略平台商贸旅游合作推介会。与土耳其、俄罗斯、吉尔吉斯斯坦部分城市签订友好交流协议。与西安市、厦门市和青岛国际港务区签署战略合作框架协议。兰州国际港务区加快建设。全年共执行招商引资项目1390个，到位资金1870.05亿元，增长38.52%。第二十届兰洽会暨民企陇上行活动引资1350.08亿元，增长16.58%。实现进出口总额45.6亿美元，与中西亚国家贸易额达到6.23亿美元。

　　一年来，我们大力转变工作作风，进一步提升工作效能。自觉接受市人大及其常委会的监督，多渠道开展民主协商，办结人大代表意见建议212件、政协委员提案343件，向市人大提交法规议案4件，制定政府规章6件。扎实开展党的群众路线教育实践活动，严肃认真查摆问题，立改立行转变作风，治污染、增收入、控物价和整治黄河风情线等一批事关民生的突出问题得到有效解决。严格落实中央八项规定和国务院“约法三章”，全市性会议压减21.43%，发文数量压缩21.3%，“三公”经费支出下降38.19%。全面停止新建楼堂馆所，市级部门单位清理腾退办公用房1.57万平方米。对33个单位、107名领导干部进行了行政问责，全市效能问责409人。按照省委党风廉政建设“3783”主体责任体系和市委《实施办法》，制定了市政府党组《实施细则》，明确重点任务，层层传导压力，努力做到“两手抓两手硬、双促进双落实”。

　　各位代表！过去一年，我市经济发展的结构、质量和效益持续向好，主要经济指标增速在全省的排名大幅提升，取得了来之不易的成绩。这是省委、省政府和市委坚强领导的结果，是市人大、市政协监督支持的结果，是全市人民共同奋斗和各方面关心帮助的结果。在此，我代表市人民政府，向全市人民、各位人大代表和政协委员、各民主党派、工商联、人民团体以及社会各界人士，向驻兰解放军指战员、武警官兵和公安民警，向所有关心和支持兰州发展的朋友们表示衷心的感谢和崇高的敬意！

　　各位代表！需要说明的是，去年我市5项经济指标与市十五届人大四次会议确定的目标有一定差距，生产总值增速欠1.6个百分点，固定资产投资增速欠4.7个百分点，社会消费品零售总额增速欠1.3个百分点，城镇居民人均可支配收入增速欠0.1个百分点，农民人均纯收入增速欠1.6个百分点。分析原因，客观上受宏观经济下行影响，投资和消费需求支撑不足，重点企业生产大幅减产；主观上是年初对形势的研判不准，对经济发展新常态认识不足，部分工作指导不够精准，组织协调和服务保障不够到位。

　　去年4月11日，我市发生局部自来水苯超标事件，给部分市民正常生活带来影响。事件发生后，我们第一时间启动应急机制，在污染源排查、饮用水供应保障、物价控制、事故调查等方面采取综合措施，使事件得到有效控制，并及时查明原因，对相关责任人员依法依规进行了问责，委托中国安全生产科学研究院对事件应急处置工作进行全面评估，形成了第三方评估报告。这一事件教训极为深刻，暴露出职能部门监管上的缺失，也影响了“五城联创”工作，需要我们认真反思，深刻吸取教训，不断强化城市公共安全管理。

　　此外，经济社会发展和政府工作中还存在一些突出问题：一是新的经济增长点处于培育壮大之中，调整经济结构、转变发展方式还需要狠下功夫；二是财政减收因素增多，刚性支出增加，收支矛盾加剧，惠民生的能力亟待提高；三是实施创新驱动战略的实际举措和平台搭建不够完善，科技人才的比较优势没有充分发挥出来；四是城市建设仍处在攻坚期，轨道交通等重大基础设施建设周期较长，给广大市民出行带来诸多不便；五是一些政府部门的“四风”问题整改不彻底，行政不作为、慢作为等现象时有发生，甚至一些政府工作人员发生违纪违规和腐败行为。对这些问题，我们将以高度负责的态度、敢于担当的精神，一项一项认真解决，真正让广大群众看到变化、见到实效、得到实惠。

　　二、2015年工作总体部署

　　今年是全面深化改革的关键之年，是全面推进依法治国的开局之年，也是全面完成“十二五”规划的收官之年。随着我国经济发展进入新常态，兰州发展面临许多新的机遇和挑战。政府工作的总体要求是：深入贯彻落实党的十八大、十八届三中、四中全会和习近平总书记系列重要讲话精神，按照中央和省市委全会暨经济工作会议部署要求，准确把握经济发展新常态，坚持稳中求进工作总基调，强化改革推动、创新驱动和城乡联动，着力深化重点领域改革、全面推进依法治市，着力加快产业转型升级、培育壮大新的经济增长极，着力保障和改善民生、加快发展各项社会事业，着力增强城市综合承载功能、扎实推进城乡一体发展，着力扩大对外开放、深度融入丝绸之路经济带建设，更好发挥中心带动作用，努力提升全市人民的幸福指数。

　　经济社会发展的主要预期目标是：生产总值增长8%以上；第一产业增加值增长6%；第二产业增加值增长7%，其中规模以上工业增加值增长6%；第三产业增加值增长10%；固定资产投资增长20%；社会消费品零售总额增长12%；公共财政预算收入增长10%；城镇居民人均可支配收入增长11%；农民人均纯收入增长12%；城镇登记失业率控制在4%以内；居民消费价格涨幅控制在3%以内。节能减排等约束性指标超额完成国家和省上下达的目标任务。

　　确定以上预期目标，把握了经济发展新常态的要求，考虑了我市转型发展的潜能和支撑，统筹了完成“十二五”规划的实际。为顺利实现今年发展的目标任务，为做大做强做美兰州进一步夯实基础，必须抓住机遇、增强信心，迎难而上、主动作为。

　　我们要深刻认识新常态的内涵、特征和深远影响，科学把握兰州所处的发展阶段和外部环境，自觉适应和积极顺应新常态，扭住发展不放松，以提高发展的质量和效益为中心，做到调速不减势、量增质更优。

　　我们要扬长补短、趋利避害，以全面深化改革为强大动力，统筹解决好发展不足和发展质量不高两大历史课题，科学谋划好发展要务和民生要事，坚定不移走以人为本、内涵式、可持续的发展路子。

　　我们要处理好市场和政府的关系，把主要精力和工作重心放到调整经济结构上来，在扩大投资带动作用的同时，更加注重挖掘和放大消费潜力，以新的增长点培育引领新的消费点，以新的消费点助推升级新的增长点，努力实现更有活力的发展。

　　我们要立足兰州新区向西开放战略平台，发挥综合保税区、兰州国际港务区的综合功能作用，构建对外承接产业转移、对内激发内生活力的发展格局，提高开放型经济发展水平。

　　我们要自觉运用法治思维、法治方式推进政府各项工作，规范权力运行，提高办事效率，维护公平正义，努力创造良好的社会环境和发展环境。

　　三、2015年主要任务

　　今年，着重做好七个方面的工作。

　　（一）以市场化为导向，着力深化重点领域改革。

　　加快转变政府职能。坚持简政放权、放管结合，完善政府部门权力清单和企业投资项目管理负面清单，制定政府部门“职责清单”。在市政务服务中心推行行政审批一口受理、规范办理和网上办理。组建兰州新区行政审批局，探索实现“一颗印章管审批”。推进近郊四区城市管理综合执法试点，确定一个区进行市场监管综合执法试点。改革公务用车制度，规范管理办公用房。

　　加大经济领域改革力度。积极推行上海自贸试验区可复制改革试点经验，争取设立中国（兰州）自由贸易区。完善财政预算管理，防范和化解政府债务风险，清理规范各类财税优惠政策。激活金融市场活力，推动政府融资平台转型发展，积极推广PPP投融资模式。加快组建兰州农商银行。深化市属国资国企改革，完善国资监管体系和公司法人治理结构，整合市属国有资产股权。鼓励社会资本参与国企改革，发展混合所有制经济。深化农村改革,规范有序推进农村土地经营权流转，搭建产权流转交易平台。

　　统筹实施其他各项改革。建立健全协调联动机制，充分发挥国家级和省级各项政策平台功能作用。落实国家考试招生制度改革政策，完善优质普通高中对口招生举措，探索义务教育集团化办学试点。开展公益性文化事业单位分类改革。加快县级公立医院综合改革。推动体育健身与医疗、文化等融合发展，支持社会力量兴办面向大众的体育健身场所设施。

　　（二）以提高质量效益为中心，着力加快产业转型升级。

　　大力发展电子信息、生物医药、新能源、新材料、节能环保等战略性新兴产业。加快推进三维数字软件产业园、四联集团西北产业基地建设，引进落地联想科技城，共建阿里飞天云大数据项目，加快3D打印和大数据产业发展。以生物产业基地建设为龙头，建成投产陇神戎发现代中药生产基地、清真阿敏生物产业园等重点项目。建成五〇四厂四期第一模块工程，开工建设汉能集团分布式薄膜太阳能项目。力争正威铜加工项目建成投产，促进科天化工达产达标。抓好红古再生资源产业园建设，创建国家城市矿产示范基地。年内新培育国家级高新技术企业10家以上。

　　促进传统产业改造升级。抓好兰石特种车辆改装、北车高端装备制造及维修、广汇煤炭分质利用、皋兰黑石川铸造产业园等重点工业项目。推进连海地区煤电冶一体化和铝深加工项目建设。抓好全国节能减排财政政策综合示范城市创建工作，扎实推进25个典型示范项目建设。培植循环经济新亮点，完成榆中和平等6个省级以上园区循环化改造，建设11条循环经济产业链，通过全省循环经济示范基地验收。加快九州“主食厨房”食品加工园区建设，促进中小食品加工企业向园区集聚发展。

　　加快现代服务业发展。开展“第三产业发展年”活动，着力发展电子商务、文化旅游、现代物流、总部经济和新兴业态。创建国家电子商务示范城市，优化提升三维商城、秀宝网等电商平台功能，推动电子商务向农村延伸。全面实施《兰州都市文化产业区规划纲要》及17个专项规划和6个配套政策，支持兰州创意文化产业园做大做强，规划建设百里黄河风情线和兴隆山两个大景区，加快黄河母亲文化公园、黄河楼、大兰山、达川三江口、河口古镇、仁寿山、天斧沙宫、什川古梨园、青城古镇等特色景点建设。优化城市商贸物流布局，打造和平-定远、水阜-忠和、东川-河口南、沙九等专业化、特色化商贸物流功能区。加快红楼时代、名城广场、兰州中心、西站中央商务区等项目建设。培育发展健康养生、体育健身、文化娱乐等新兴业态，加快形成多点支撑的消费格局。

　　深入实施创新驱动战略。以深化科技体制改革为主线，以提高区域创新能力为核心，推进兰白科技创新改革试验区建设。加强与上海张江自主创新示范区在人才、技术、信息、产业开发等方面合作，引进一批项目在兰州转化、落地、生产。把孵化器作为产学研融合的突破口，加快完善电子信息、生物医药和“中国兰州留学人员创业园彭家坪产业研发基地”三个科技孵化器的运行机制。建成运营兰州科技大市场。继续办好兰州科技成果交易周。积极发展科技中介咨询服务。设立科技创新投资基金和创业投资引导基金，支持研发攻关、成果转化、中试孵化和创新创业，吸引更多创新型人才到兰州创业发展。

　　（三）以兰州新区为战略平台，着力培育壮大新的经济增长极。进一步明确三个国家级开发区功能定位，推进兰州新区综合配套改革试验区建设，形成功能互补、错位发展的格局。

　　加快综合保税区建设。建成保税区综合服务大楼、海关国检查验用房、配套公寓和信息化等工程，确保通过国家验收并封关运营。坚持优中选优，以物流类和两头在外的加工企业为重点，精准引进一批高科技、低碳环保企业，力争一批项目开工建设。完善管理机制，吸引高端人才，建立富有活力的运营模式。

　　着力培育壮大六大产业链。坚持引高、引强、引新、引导，优化产业园区布局，加大招商引资力度，承接东部产业转移，打造石油化工、装备制造、电子信息、新材料、光电制造、生物医药六大产业链。推动市州“飞地经济”产业园建设。加快推进工业企业“出城入园”，力争33个在建项目建成投产、38个项目开工建设。争取中国电信、中国联通等在新区设立大数据中心，引进中星微电子、中兴通讯等一批项目。推进兰州新区科技创新城建设，力争北京大学在新区设立技术转移中心。

　　继续完善基础设施功能。新建33条、续建85条主次干道，新区道路通车里程达到300公里以上。加快拓展对外交通，建成兰州至中川城际铁路，力争开工建设市区至新区中通道，启动国道341线中川至永登段工程。推进水、电、气、暖、垃圾污水处理、通讯、防洪等设施建设。

　　强化公共服务体系建设。坚持以优质的教育、卫生、商贸资源吸引和集聚人气，建成兰炼一小、兰州十一中新区分校和中小学综合
